# Characterization of magnetic nanoparticle by dynamic light scattering

**DOI:** 10.1186/1556-276X-8-381

**Published:** 2013-09-08

**Authors:** JitKang Lim, Swee Pin Yeap, Hui Xin Che, Siew Chun Low

**Affiliations:** 1 School of Chemical Engineering, Universiti Sains Malaysia, Nibong Tebal, Penang, 14300, Malaysia; 2 Department of Physics, Carnegie Mellon University, Pittsburgh, PA, 15213, USA

**Keywords:** Dynamic light scattering, Magnetic nanoparticles, Colloidal stability, Surface functionalization, Review

## Abstract

Here we provide a complete review on the use of dynamic light scattering (DLS) to study the size distribution and colloidal stability of magnetic nanoparticles (MNPs). The mathematical analysis involved in obtaining size information from the correlation function and the calculation of *Z*-average are introduced. Contributions from various variables, such as surface coating, size differences, and concentration of particles, are elaborated within the context of measurement data. Comparison with other sizing techniques, such as transmission electron microscopy and dark-field microscopy, revealed both the advantages and disadvantages of DLS in measuring the size of magnetic nanoparticles. The self-assembly process of MNP with anisotropic structure can also be monitored effectively by DLS.

## Review

### Introduction

Magnetic nanoparticles (MNPs) with a diameter between 1 to 100 nm have found uses in many applications [1,2]. This nanoscale magnetic material has several advantages that provide many exciting opportunities or even a solution to various biomedically [3-5] and environmentally [6-8] related problems. Firstly, it is possible to synthesize a wide range of MNPs with well-defined structures and size which can be easily matched with the interest of targeted applications. Secondly, the MNP itself can be manipulated by an externally applied magnetic force. The capability to control the spatial evolution of MNPs within a confined space provides great benefits for the development of sensing and diagnostic system/techniques [9,10]. Moreover MNPs, such as Fe^0^ and Fe_3_O_4_, that exhibit a strong catalytic function can be employed as an effective nanoagent to remove a number of persistent pollutants from water resources [11,12]. In addition to all the aforementioned advantages, the recent development of various techniques and procedures for producing highly monodispersed and size-controllable MNPs [13,14] has played a pivotal role in promoting the active explorations and research of MNPs.

In all of the applications involving the use of MNPs, the particle size remained as the most important parameter as many of the chemical and physical properties associated to MNPs are strongly dependent upon the nanoparticle diameter. In particular, one of the unique features of a MNP is its high-surface-to-volume ratio, and this property is inversely proportional to the diameter of the MNP. The smaller the MNP is, the larger its surface area and, hence, the more loading sites are available for applications such as drug delivery and heavy metal removal. Furthermore, nanoparticle size also determines the magnetophoretic forces (*F*_mag_) experienced by a MNP since *F*_mag_ is directly proportional to the volume of the particles [15]. In this regard, having size information is crucial as at nanoregime, the MNP is extremely susceptible to Stoke’s drag [16] and thermal randomization energy [17]. The successful manipulation of MNP can only be achieved if the *F*_mag_ introduced is sufficient to overcome both thermal and viscous hindrances [18]. In addition, evidences on the (eco)toxicological impacts of nanomaterials have recently surfaced [19]. The contributing factors of nanotoxicity are still a subject of debate; however, it is very likely due to either (1) the characteristic small dimensional effects of nanomaterials that are not shared by their bulk counterparts with the same chemical composition [20] or (2) biophysicochemical interactions at the nano-bio interface dictated by colloidal forces [21]. For either reason, the MNP’s size is one of the determining factors.

The technique of dynamic light scattering (DLS) has been widely employed for sizing MNPs in liquid phase [22,23]. However, the precision of the determined particle size is not completely understood due to a number of unevaluated effects, such as concentration of particle suspension, scattering angle, and shape anisotropy of nanoparticles [24]. In this review, the underlying working principle of DLS is first provided to familiarize the readers with the mathematical analysis involved for correct interpretation of DLS data. Later, the contribution from various factors, such as suspension concentration, particle shape, colloidal stability, and surface coating of MNPs, in dictating the sizing of MNPs by DLS is discussed in detail. It is the intention of this review to summarize some of the important considerations in using DLS as an analytical tool for the characterization of MNPs.

### Overview of sizing techniques for MNPs

There are numerous analytical techniques, such as DLS [25], transmission electron miscroscopy (TEM) [26], thermomagnetic measurement [27], dark-field microscopy [17,18], atomic force microscopy (AFM) [28], and acoustic spectrometry measurement [29], that have been employed to measure the size/size distribution of MNPs (Table 1). TEM is one of the most powerful analytical tools available which can give direct structural and size information of the MNP. Through the use of the short wavelengths achievable with highly accelerated electrons, it is capable to investigate the structure of a MNP down to the atomic level of detail, whereas by performing image analysis on the TEM micrograph obtained, it is possible to give quantitative results on the size distribution of the MNP. This technique, however, suffered from the small sampling size involved. A typical MNP suspension composed of 10^10^ to 10^15^ particles/mL and the size analysis by measuring thousands or even tens of thousands of particles still give a relatively small sample pool to draw statistically conclusive remarks.

**Table 1:** Common analytical techniques and the associated range scale involved for nanoparticle sizing

**Techniques**	**Approximated working size range**
Dynamic light scattering	1 nm to approximately 5 μm
Transmission electron microscopy	0.5 nm to approximately 1 μm
Atomic force microscopy	1 nm to approximately 1 μm
Dark-field microscopy	5 to 200 nm
Thermomagnetic measurement	10 to approximately 50 nm

Thermomagnetic measurement extracts the size distribution of an ensemble of superparamagnetic nanoparticles from zero-field cooling (ZFC) magnetic moment, *m*_ZFC_(T), data based on the Néel model [27]. This method is an indirect measurement of particle size and relies on the underlying assumption of the mathematical model used to calculate the size distribution. In addition, another limitation of this analytical method includes the magnetic field applied for ZFC measurements which must be small compared to the anisotropy field of the MNPs [30], and it also neglects particle-particle dipolar interactions which increase the apparent blocking temperature [31]. This technique, however, could give a very reliable magnetic size of the nanoparticle analyzed.

Dark-field microscopy relies on direct visual inspection of the optical signal emitted from the MNP while it undergoes Brownian motion. After the trajectories of each MNP over time *t* are recorded, the two-dimensional mean-squared displacement *<r*^*2*^*> =* 4*Dt* is used to calculate the diffusion coefficient *D* for each particle. Later on, the hydrodynamic diameters can be estimated via the Stokes-Einstein equation for the diffusion coefficients calculated for individual particles, averaging over multiple time steps [18]. Successful implementation of this technique depends on the ability to trace the particle optically by coating the MNP with a noble metal that exhibits surface Plasmon resonance within a visible wavelength. This extra synthesis step has significantly restricted the use of this technique as a standard route for sizing MNPs. The size of an MNP obtained through dark-field microscopy is normally larger than the TEM and DLS results [17]. It should be noted that dark-field microscopy can also be employed for direct visualization of a particle flocculation event [32]. As for AFM, besides the usual topographic analysis, magnetic imaging of a submicron-sized MNP grown on GaAs substrate has been performed with magnetic force microscopy equipment [33]. Despite all the recent breakthroughs, sample preparation and artifact observation are still the limiting aspect for the wider use of this technology for sizing MNPs [34].

The particle size and size distribution can also be measured with an acoustic spectrometer which utilizes the sound pulses transmitted through a particle suspension to extract the size-related information [29]. Based on the combined effect of absorption and scattering of acoustic energy, an acoustic sensor measures attenuation frequency spectra in the sample. This attenuation spectrum is used to calculate the particle size distribution. This technique has advantages over the light scattering method in studying samples with high polydispersity as the raw data for calculating particle size depend on only the third power of the particle size. This scenario makes contribution of the small (nano) and larger particles more even and the method potentially more sensitive to the nanoparticle content even in the very broad size distributions [35].

DLS, also known as photon correlation spectroscopy, is one of the most popular methods used to determine the size of MNPs. During the DLS measurement, the MNP suspension is exposed to a light beam (electromagnetic wave), and as the incident light impinges on the MNP, the direction and intensity of the light beam are both altered due to a process known as scattering [36]. Since the MNPs are in constant random motion due to their kinetic energy, the variation of the intensity with time, therefore, contains information on that random motion and can be used to measure the diffusion coefficient of the particles [37]. Depending on the shape of the MNP, for spherical particles, the hydrodynamic radius of the particle *R*_H_ can be calculated from its diffusion coefficient by the Stokes-Einstein equation *D*_*f*_*= k*_B_*T/*6*πηR*_H_, where *k*_B_ is the Boltzmann constant, *T* is the temperature of the suspension, and *η* is the viscosity of the surrounding media. Image analysis on the TEM micrographs gives the ‘true radius’ of the particles (though determined on a statistically small sample), and DLS provides the hydrodynamic radius on an ensemble average [38]. The hydrodynamic radius is the radius of a sphere that has the same diffusion coefficient within the same viscous environment of the particles being measured. It is directly related to the diffusive motion of the particles.

DLS has several advantages for sizing MNPs and has been widely used to determine the hydrodynamic size of various MNPs as shown in Table 2. First of all, the measuring time for DLS is short, and it is almost all automated, so the entire process is less labor intensive and an extensive experience is not required for routine measurement. Furthermore, this technique is non-invasive, and the sample can be employed for other purposes after the measurement. This feature is especially important for the recycle use of MNP with an expensive surface functional group, such as an enzyme or molecular ligands. In addition, since the scattering intensity is directly proportional to the sixth power of the particle radius, this technique is extremely sensitive towards the presence of small aggregates. Hence, erroneous measurement can be prevented quite effectively even with the occurrences of limited aggregation events. This unique feature makes DLS one of the very powerful techniques in monitoring the colloidal stability of MNP suspension.

**Table 2:** Hydrodynamic diameter of different MNPs determined by DLS

**Type of MNPs**	**Surface coating**	**Hydrodynamic diameter by DLS (nm)**	**Reference**
Fe^0^	Carboxymethyl cellulose	15-19	[39]
	Guar gum	350-700	[40]
	Poly(methacrylic acid)-poly(methyl methacrylate)-poly(styrenesulfonate) triblock copolymer	100-600	[41]
	Poly(styrene sulfonate)	30-90	[22]
γ-Fe_2_O_3_	Oleylamine or oleic acid	5-20	[42]
	Poly(*N*,*N*-dimethylacrylamide)	55-614	[43]
	Poly(ethylene oxide)-block-poly(glutamic acid)	42-68	[44]
	Poly(ethylene imine)	20-75	[45]
	Poly(ϵ-caprolactone)	193 ± 7	[46]
Fe_3_O_4_	Phospholipid-PEG	14.7 ± 1.4	[47]
	Polydimethylsiloxane	41.2 ± 0.4	[48]
	Oleic acid-pluronic	50-600	[49]
	Polyethylenimine (PEI)	50-150	[23,50]
	Polythylene glycol	10-100	[51]
	Triethylene glycol	16.5 ± 3.5	[52]
	Poly(*N*-isopropylacrylamide)	15-60	[53]
	Pluronic F127	36	[54]
	Poly(sodium 4-styrene sulfonate)	~200	[55]
	Poly(diallyldimethylammonium chloride)	107.4 ± 53.7	[56]
FePt	Poly(diallyldimethylammonium chloride)	30-100	[57]
NiO	Cetyltrimethyl ammonium bromide	10-80	[58]
	Fetal bovine serum	39.05	[59]
	Not specified	750 ± 30	[60]
CoO, Co_2_O_3_	Poly(methyl methacrylate)	59-85	[61]
CoFe	Hydroxamic and phosphonic acids	6.5-458.7	[62]

### The underlying principle of DLS

The interaction of very small particles with light defined the most fundamental observations such as why is the sky blue. From a technological perspective, this interaction also formed the underlying working principle of DLS. It is the purpose of this section to describe the mathematical analysis involved to extract size-related information from light scattering experiments.

#### The correlation function

**Figure 1:**
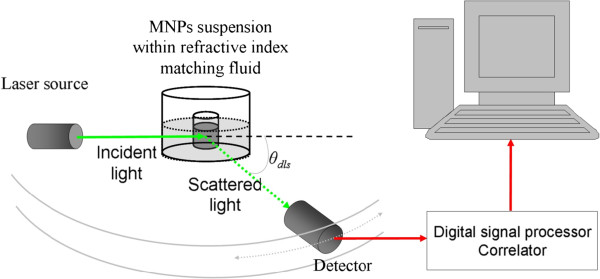
**Optical configuration of the typical experimental setup for dynamic light scattering measurements.** The setup can be operated at multiple angles.

**Figure 2:**
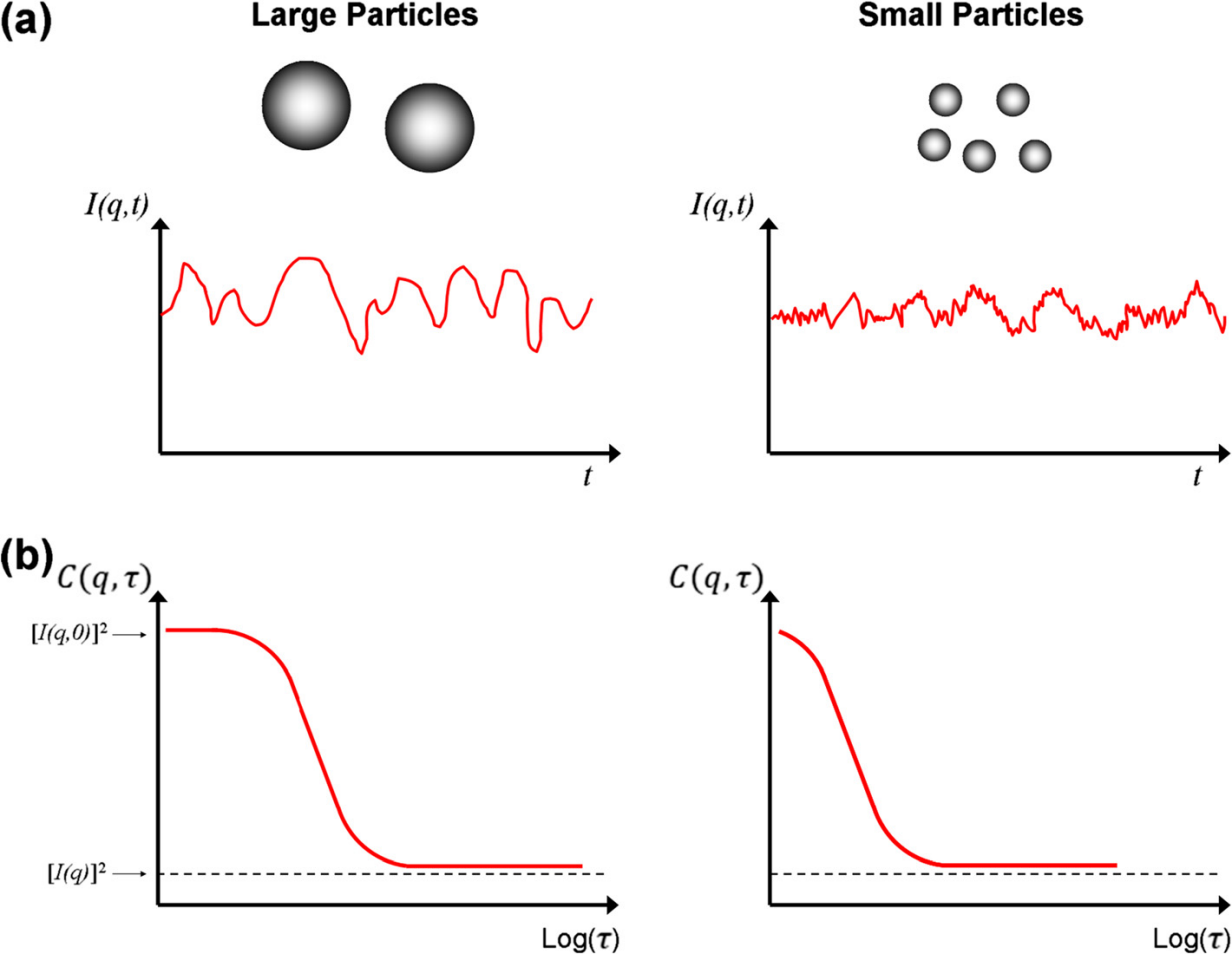
**Schematic illustration of intensity measurement and the corresponding autocorrelation function in dynamic light scattering.** The figure illustrates dispersion composed of large and small particles. **(a)** Intensity fluctuation of scattered light with time, and **(b)** the variation of autocorrelation function with delay time.

DLS measures the scattered intensity over a range of scattering angles *θ*_dls_ for a given time *t*_*k*_ in time steps ∆*t*. The time-dependent intensity *I*(*q*, *t*) fluctuates around the average intensity *I*(*q*) due to the Brownian motion of the particles [38]:

(1) Iq=limtk→∞1tk∫0tkIq,t·dt≈limk→∞1k∑i=1kIq,i·Δt

where [*I(q)*] represents the time average of *I(q)*. Here, it is assumed that *t*_*k*_, the total duration of the time step measurements, is sufficiently large such that *I(q)* represents average of the MNP system. In a scattering experiment, normally, *θ*_dls_ (see Figure 1) is expressed as the magnitude of the scattering wave vector *q* as

(2) $$q=\left(4\mathrm{\pi n}/\lambda \right)\sin\left(\theta_{\mathrm{dls}}/2\right)$$

where *n* is the refractive index of the solution and *λ* is the wavelength in vacuum of the incident light. Figure 2a illustrates typical intensity fluctuation arising from a dispersion of large particles and a dispersion of small particles. As the small particles are more susceptible to random forces, the small particles cause the intensity to fluctuate more rapidly than the large ones.

The time-dependent intensity fluctuation of the scattered light at a particular angle can then be characterized with the introduction of the autocorrelation function as

(3) cq,τ=limtk→∞1tk∫0tkIq,t·Iq,t+τ·dt≈limk→∞1k∑j=0kIq,i·Δt·Iq,i+j·Δt

where *τ* = *i* ∆*t* is the delay time, which represents the time delay between two signals *I*(*q*,*i Δt*) and *I*(*q*,(*i* + *j*) *Δt*). The function *C*(*q*,*τ*) is obtained for a series of *τ* and represents the correlation between the intensity at *t*_1_ (*I*(*q*,*t*_1_)) and the intensity after a time delay of *τ* (*I*(*q*,*t*_1_ + *τ*)). The last part of the equation shows how the autocorrelation function is calculated experimentally when the intensity is measured in discrete time steps [37]. As for nanoparticle dispersion, the autocorrelation function decays more rapidly for small particles than for the large particles as depicted in Figure 2b. The autocorrelation function has its highest value of [*I*(*q*,*0*)]^2^ at *τ* = 0. As *τ* becomes sufficiently large at long time scales, the fluctuations becomes uncorrelated and *C*(*q*,*τ*) decreases to [*I*(*q*)]^2^. For non-periodic *I*(*q*,*t*), a monotonic decay of *C*(*q*,*τ*) is observed as *τ* increases from zero to infinity and

(4) Cq,τIq2=g2q,τ=1+ξg1q,τ2

where *ξ* is an instrument constant approximately equal to unity and *g*^(1)^(*q*,*τ*) is the normalized electric field correlation function [63]. Equation 4 is known as the Siegert relation and is valid except in the case of scattering volume with a very small number of scatterers or when the motion of the scatterers is limited. For monodisperse, spherical particles, *g*^(1)^(*τ*) is given by

$$g^{\left(1\right)}\left(q,\tau \right)=\exp\left(-D_{f}q^{2}\tau \right).$$

Once the value of *D*_*f*_ is obtained, the hydrodynamic diameter of a perfectly monodisperse dispersion composed of spherical particles can be inferred from the Stokes-Einstein equation. Practically, the correlation function observed is not a single exponential decay but can be expressed as

(6) $$g^{\left(1\right)}\left(q,\tau \right)=\int_{0}^{\infty }G\left(\Gamma \right)e^{-\Gamma \tau }\mathrm{d\Gamma}$$

where *G*(*Γ*) is the distribution of decay rates *Γ*. For a narrowly distributed decay rate, cumulant method can be used to analyze the correlation function. A properly normalized correlation function can be expressed as

(7) $$\ln\left(g^{\left(1\right)}\left(q,\tau \right)\right)=-\langle \Gamma \rangle \tau +\frac{\mu_{2}}{2}\tau^{2}$$

where 〈*Γ*〉 is the average decay rate and can be defined as

(8) $$\langle \Gamma \rangle =\int_{0}^{\infty }G\left(\Gamma \right)\mathrm{\Gamma d\Gamma}$$

and *μ*_2_ = 〈*Γ*〉^2^ − 〈*Γ*〉^2^ is the variance of the decay rate distribution. Then, the polydispersity index (PI) is defined as PI = μ_2_/〈*Γ*〉^2^. The average hydrodynamic radius is obtained from the average decay rate 〈*Γ*〉 using the relation

(9) $$R_{H}=\frac{k_{B}T}{6\pi \eta \langle \Gamma \rangle }q^{2}$$

#### Z-average

In most cases, the DLS results are often expressed in terms of the *Z*-average. Since the *Z*-average arises when DLS data are analyzed through the use of the cumulant technique [64], it is also known as the “cumulant mean.” Under Rayleigh scattering, the amount of light scattered by a single particle is proportional to the sixth power of its radius (volume squared). This scenario causes the averaged hydrodynamic radius determined by DLS to be also weighted by volume squared. Such an averaged property is called the *Z*-average. For particle suspension with discrete size distribution, the *Z*-average of some arbitrary property *y* would be calculated as

(10) $$\langle y\rangle =\frac{\Sigma_{i}n_{i}R_{H,i}^{6}y_{i}}{\Sigma_{i}n_{i}R_{H,i}^{6}}$$

where *n*_*i*_ is the number of particles of type *i* having a hydrodynamic radius of *R*_H,*i*_ and property *y*. If we assume that this particle dispersion consists of exactly two sizes of particles 1 and 2, then Equation 10 yields

(11) $$\langle y\rangle =\frac{n_{1}R_{H,1}^{6}y_{1}+n_{2}R_{H,2}^{6}y_{2}}{n_{1}R_{H,1}^{6}+n_{2}R_{H,2}^{6}}$$

where *R*_H,*i*_ and *y*_*i*_ are the volume and arbitrary property for particle 1 (*i* = 1) and particle 2 (*i* = 2). Suppose that two particles 1 combined to form one particle 2 and assume that we start with *n*_0_ total of particle 1, some of which combined to form *n*_2_ number of particle 2. With this assumption, we have *n*_1_ = *n*_0_*– n*_2_ number of particle 1. Moreover, under this assumption *R*_H,2_ = 2 *R*_H,1_. Substitute these relations into Equation 11; then, the *Z*-average of property *y* becomes

(12) $$\frac{\langle y\rangle }{y_{1}}=\frac{1+\left(2\left(\frac{y_{2}}{y_{1}}\right)-1\right)2\left(\frac{n_{2}}{n_{0}}\right)}{1+2\left(\frac{n_{2}}{n_{0}}\right)}$$

where *2n*_2_/*n*_0_ is the fraction of total particle 1 existing as particle 2. Solving this fraction, we obtained

(13) $$\frac{2n_{2}}{n_{0}}=\frac{\frac{\langle y\rangle }{y_{1}}-1}{\frac{2y_{2}}{y_{1}}-\frac{\langle y\rangle }{y_{1}}-1}$$

**Figure 3:**
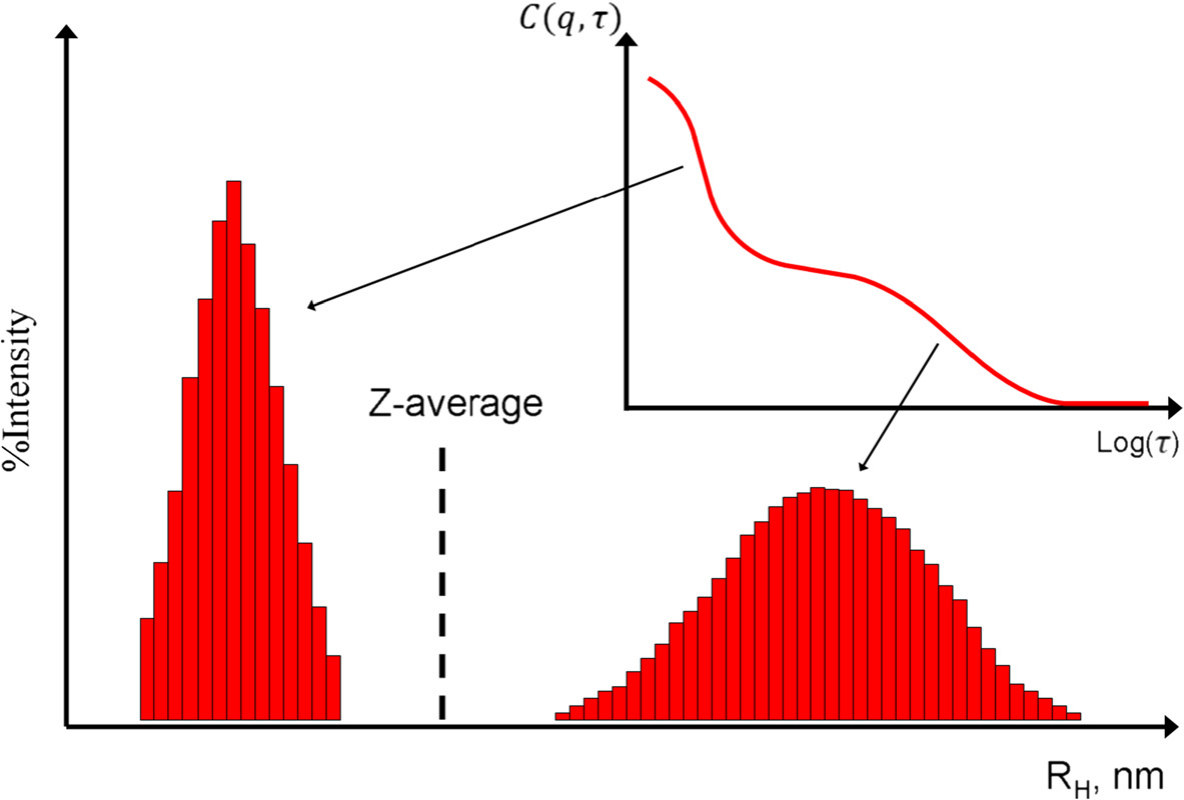
***Z*-average (cumulant) size for particle suspension with bimodal distribution.**

However, it should be noted that *Z*-average should only be employed to provide the characteristic size of the particles if the suspension is monomodal (only one peak), spherical, and monodisperse. As shown in Figure 3, for a mixture of particles with obvious size difference (bimodal distribution), the calculated *Z*-average carries irrelevant size information.

### DLS measurement of MNPs

**Figure 4:**
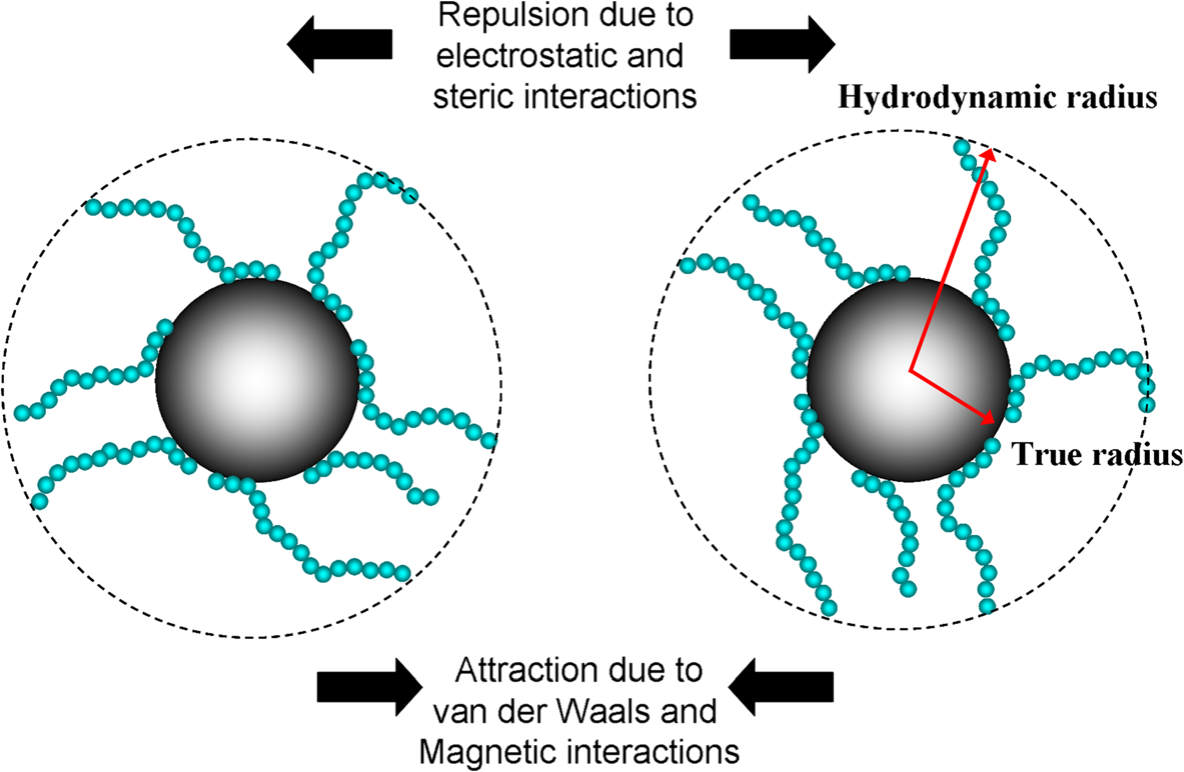
**Pictorial representation of two MNPs and major interactions.** The image shows two MNPs coated with macromolecules with repeated segments and the major interactions involved between them in dictating the colloidal stability of MNP suspension.

The underlying challenges of measuring the size of MNPs by DLS lay in the facts that (1) for engineering applications, these particles are typically coated with macromolecules to enhance their colloidal stability (see Figure 4) and (2) there present dipole-dipole magnetic interactions between the none superparamagnetic nanoparticles. Adsorbing macromolecules onto the surface of particles tends to increase the apparent *R*_H_ of particles. This increase in *R*_H_ is a convenient measure of the thickness of the adsorbed macromolecules [65]. This section is dedicated to the scrutiny of these two phenomena and also suspension concentration effect in dictating the DLS measurement of MNPs. All DLS measurements were performed with a Malvern Instrument Zetasizer Nano Series (Malvern Instruments, Westborough, MA, USA) equipped with a He-Ne laser (*λ* = 633 nm, max 5 mW) and operated at a scattering angle of 173°. In all measurements, 1 mL of particle suspensions was employed and placed in a 10 mm × 10 mm quartz cuvette. The iron oxide MNP used in this study was synthesized by a high-temperature decomposition method [17].

#### Size dependency of MNP in DLS measurement

**Figure 5:**
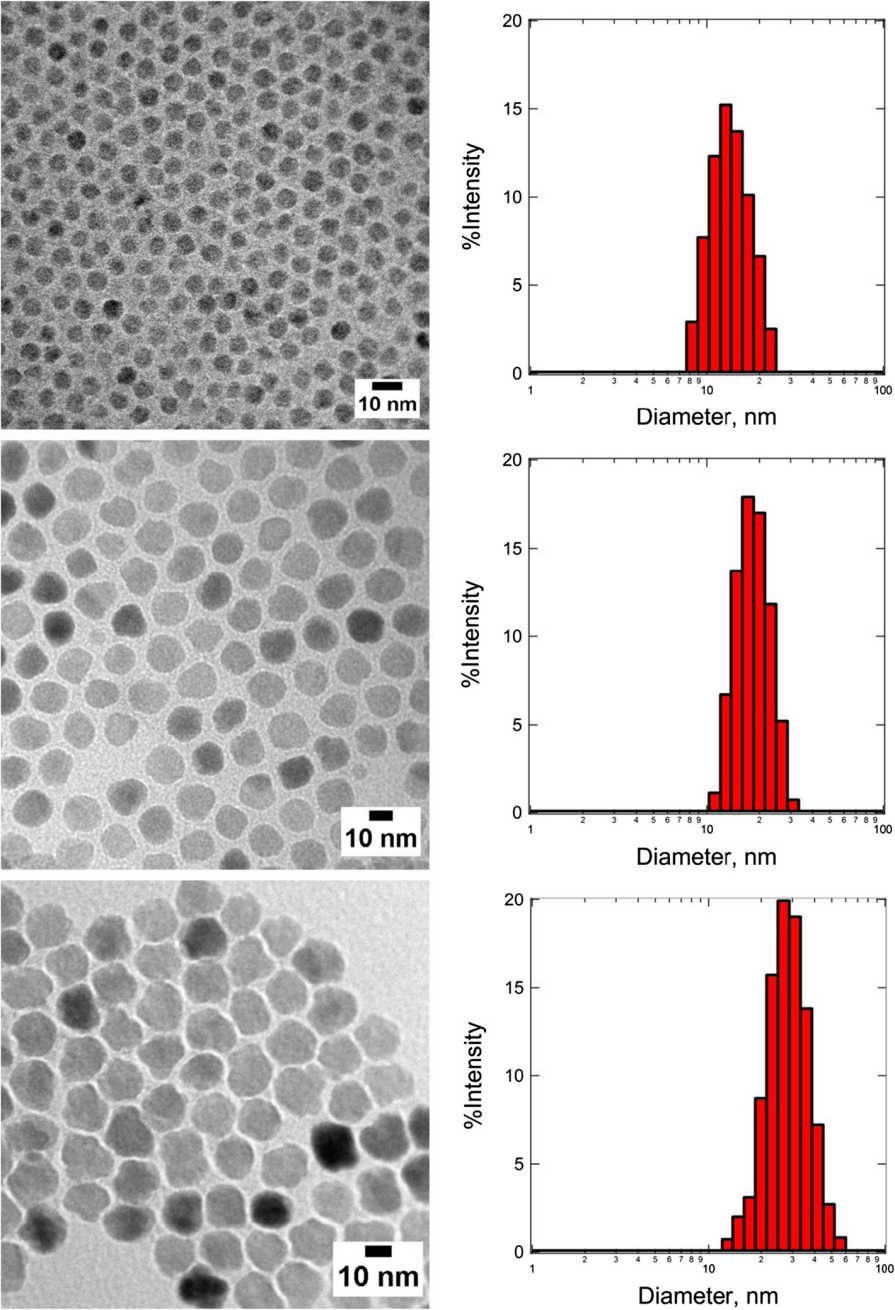
**TEM micrographs of Fe**_**3**_**O**_**4 **_**MNPs with their size distribution determined by DLS.** The *Z*-average of MNP calculated from the DLS data is (top) 16.9 ± 5.2 nm, (middle) 21.1 ± 5.5 nm, and (bottom) 43.1 ± 14.9 nm, respectively.

In order to demonstrate the sizing capability of DLS, measurements were conducted on three species of Fe_3_O_4_ MNPs produced by high-temperature decomposition method which are surface modified with oleic acid/oleylamine in toluene (Figure 5). The TEM image analyses performed on micrographs shown in Figure 5 (from top to bottom) indicate that the diameter of each particle species is 7.2 ± 0.9 nm, 14.5 ± 1.8 nm, and 20.1 ± 4.3 nm, respectively. The diameters of these particles obtained from TEM and DLS are tabulated in Table 3. It is very likely that the main differences between the measured diameters from these two techniques are due to the presence of an adsorbing layer, which is composed of oleic acid (OA) and oleylamine (OY), on the surface of the particle. Small molecular size organic compounds, such as OA and OY, are electron transparent, and therefore, they did not show up in the TEM micrograph (Figure 5). Given that the chain lengths of OA and OY are approximately 2 nm [66,67], the best match of DLS and TEM, in terms of measured diameter, can be observed from middle-sized Fe_3_O_4_ MNPs.

**Table 3:** **Diameter of Fe**_**3**_**O**_**4 **_**MNP determined by TEM and DLS ( *Z *-average)**

**Particle**	**TEM (nm)**	**DLS (nm)**	**Difference (nm)**
Fe_3_O_4_	7.2	16.9	9.7
	14.5	21.1	6.6
	20.1	43.1	23.0

For small-sized MNPs, the radius of curvature effect is the main contributing factor for the large difference observed on the averaged diameter from DLS and TEM. This observation has at least suggested that for any inference of layer thickness from DLS measurement, the particles with a radius much larger than the layer thickness should be employed. In this measurement, the fractional error in the layer thickness can be much larger than the fractional error in the radius with the measurement standard deviation of only 0.9 nm for TEM but at a relatively high value of 5.2 nm for DLS. At a very large MNP size of around 20 nm (bottom image of Figure 5), the *Z*-average hydrodynamic diameter is 23 nm larger than the TEM size. Moreover, the standard deviation of the DLS measurement of this particle also increased significantly to 14.9 nm compared to 5.2 and 5.5 nm for small- and middle-sized MNPs, respectively. This trend of increment observed in standard deviation is consistent with TEM measurement. Both the shape irregularity and polydispersity, which are the intrinsic properties that can be found in a MNP with a diameter of 20 nm or above, contribute to this observation. For a particle larger than 100 nm, other factors such as electroviscous and surface roughness effects should be taken into consideration for the interpretation of DLS results [68].

#### MNP concentration effects

In DLS, the range of sample concentration for optimal measurements is highly dependent on the sample materials and their size. If the sample is too dilute, there may be not enough scattering events to make a proper measurement. On the other hand, if the sample is too concentrated, then multiple scattering can occur. Moreover, at high concentration, the particle might not be freely mobile with its spatial displacement driven solely by Brownian motion but with the strong influences of particle interactions. This scenario is especially true for the case of MNPs with interparticle magnetic dipole-dipole interactions.

**Figure 6:**
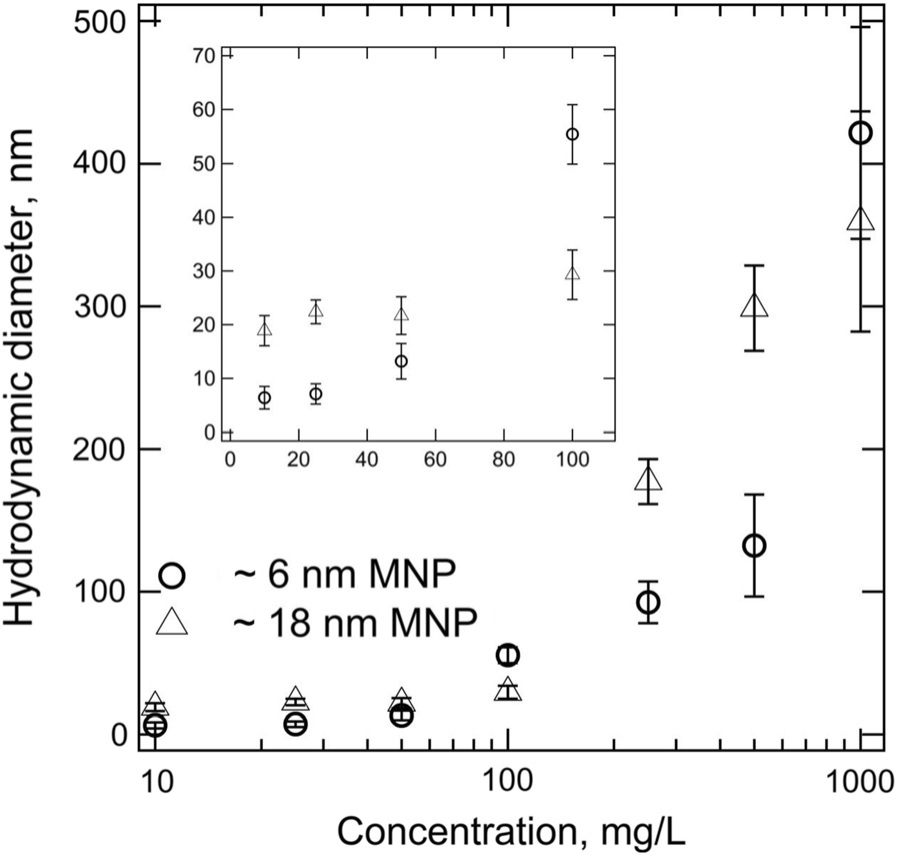
Particle concentration effects on the measurement of hydrodynamic diameter by DLS.

Figure 6 illustrates the particle concentration effects on 6- and 18-nm superparamagnetic iron oxide MNPs, with no surface coating, dispersed in deionized water. Both species of MNPs show strong concentration dependency as their hydrodynamic diameter increases with the concentration increment. The hydrodynamic diameter for small particles increases from 7.1 ± 1.9 nm to 13.2 ± 3.3 nm as the MNP concentration increases from 25 to 50 mg/L. On the other hand, the hydrodynamic diameter of large particles remains to be quite constant until around 100 mg/L and then only experiences a rapid jump of the detected size from 29.3 ± 4.6 nm (at 100 mg/L) to 177.3 ± 15.8 nm (at 250 mg/L). Since the concentration of the MNP is prepared in mass basis, the presence of an absolute number of particles in a given volume of solution is almost two orders of magnitude higher in a small-particle suspension. For example, at 100 mg/L, the concentrations for small and larger particles are calculated as 1.7 × 10^20^ particles (pts)/m^3^ and 6.3 × 10^18^ pts/m^3^ by assuming that the composition material is magnetite with a density of 5.3 g/cm^3^. This concentration translated to a collision frequency of 85,608 s^−1^ and 1,056 s^−1^. So, at the same mass concentration, it is more likely for small particles to experience the non-self-diffusion motions.

For both species of particles, the upward trends of hydrodynamic diameter, which associates to the decrement of diffusion coefficient, reflect the presence of a strong interaction between the particles as MNP concentration increases. Furthermore, since the aggregation rate has a second-order dependency on particle concentration [69], the sample with high MNP concentration has higher tendency to aggregate, leading to the formation of large particle clusters. Therefore, the initial efforts for MNP characterization by using DLS should focus on the determination of the optimal working concentration.

#### Colloidal stability of MNPs

**Figure 7:**
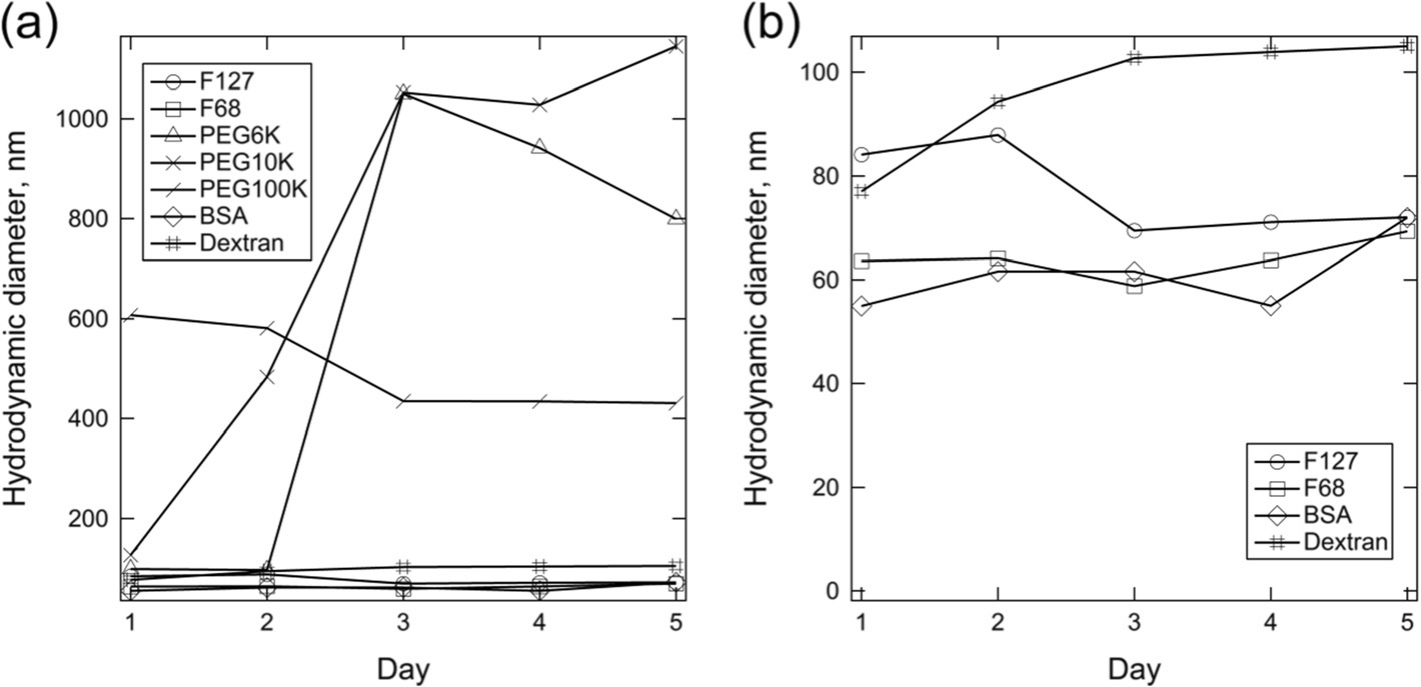
**Intensity-weighted average hydrodynamic diameter for core-shell nanoparticles with different adsorbed macromolecules in PBS. (a)** Extensive aggregation is evident with PEG 6k, PEG10k, and PEG100k, while **(b)** bovine serum albumin (BSA), dextran, Pluronic F127, and Pluronic F68 provided stable hydrodynamic diameters over the course of 5 days. ‘Day 0’ corresponds to the start of the overnight adsorption of macromolecules to the MNPs. Copyright 2009 American Chemical Society. Reprinted with permission from [65].

Another important use of DLS in the characterization of MNPs is for monitoring the colloidal stability of the particles [70]. An iron oxide MNP coated with a thin layer of gold with a total diameter of around 50 nm is further subjected for surface functionalization by a variety of macromolecules [65]. The colloidal stability of the MNP coated with all these macromolecules suspended in 154 mM ionic strength phosphate buffer solution (PBS) (physiologically relevant environment for biomedical application) is monitored by DLS over the course of 5 days (Figure 7). The uncoated MNP flocculated immediately after their introduction to PBS and is verified with the detection of micron-sized objects by DLS.

As shown in Figure 7, both polyethylene glycol (PEG) 6k and PEG 10k are capable of tentatively stabilizing the MNPs in PBS for the first 24 and 48 h. Aggregation is observed with the detection of particle clusters with a diameter of more than 500 nm. After this period of relative stability, aggregation accelerated to produce micron-sized aggregates by day 3. Actually, the continuous monitoring of MNP size by DLS after this point is less meaningful as the dominating motion is the sedimentation of large aggregates [71]. For PEG 6k and PEG 10k that have a rather low degree of polymerization, the loss of stability over a day or two could have been due to slow PEG desorption that would not be expected of larger polymers. Nevertheless, PEG 100k-coated MNPs were not as well stabilized as the PEG 6k- or PEG 10k-coated ones, despite the higher degree of polymerization that one might expect to produce greater adsorbed layer thicknesses and therefore longer-ranged steric forces. In addition to the degree of polymerization, as discussed by Golas and coworkers [72], the colloidal stability of polymeric stabilized MNPs is also dependent on other structural differences of the polymer employed, such as the chain architecture and the identity of the charged functional unit. In their work, DLS was used to confirm the nanoparticle suspensions that displayed the least sedimentation which was indeed stable against aggregation.

**Figure 8:**
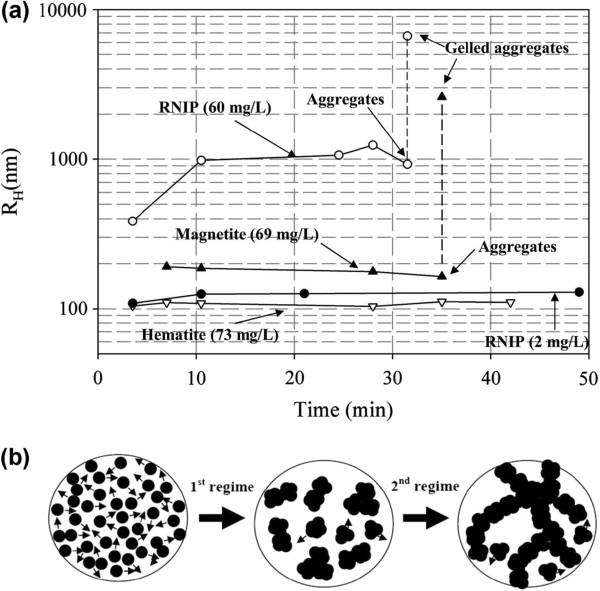
**Evolution of hydrodynamic radius and MNP aggregation and gelation. (a)** Evolution of the average hydrodynamic radius of dominant size class of MNPs as a function of time for RNIP (Fe^0^/Fe_3_O_4_), magnetite, and hematite at pH 7.4. The particle size distribution for RNIP and magnetite becomes bimodal at the last measured point due to gelation of aggregates. **(b)** Rapid MNP aggregation and subsequent chain-like gelation: rapid aggregation of MNP to form micron-sized clusters (first regime) and chain-like aggregation and gelation of the micron-sized aggregates (second regime). Copyright 2007 American Chemical Society. Reprinted with permission from [73].

In addition to the popular use of DLS in sizing individual MNPs, this analytical technique is also being employed to monitor the aggregation behavior of MNPs and the size of final clusters formed [55,73]. The study of particle aggregates is important since the magnetic collection is a cooperative phenomenon [74,75]. Subsequently, it is much easier to harvest submicron-sized MNP clusters than individual particles. Hence, a magnetic nanocluster with loss-packed structure and uniform size and shape has huge potential for various engineering applications in which the real-time separation is the key requirement [76]. Therefore, the use of DLS to monitor the aggregation kinetic of MNPs is important to provide direct feedback about the time scale associated with this process [55,77]. Figure 8 illustrates the aggregation behavior of three species of 40-nm reactive nanoscale iron particles (RNIP), 27.5-nm magnetite (Fe_3_O_4_) MNP, and 40-nm hematite (α-Fe_2_O_3_) MNP [73]. Phenrat and coworkers have demonstrated that DLS can be an effective tool to probe the aggregation behavior of MNPs (Figure 8a). The time evolution of the hydrodynamic radius of these particles from monomodal to bimodal distribution revealed the aggregation kinetic of the particles. Together with the in situ optical microscopy observation, the mechanism of aggregation is proposed as the transitions from rapidly moving individual MNPs to the formation of submicron clusters that lead to chain formation and gelation (Figure 8b). By the combination of small-angle neutron scattering and cryo-TEM measurements, DLS can also be used as an effective tool to understand the fractal structure of this aggregate [78].

### DLS measurement of non-spherical MNPs

**Figure 9:**
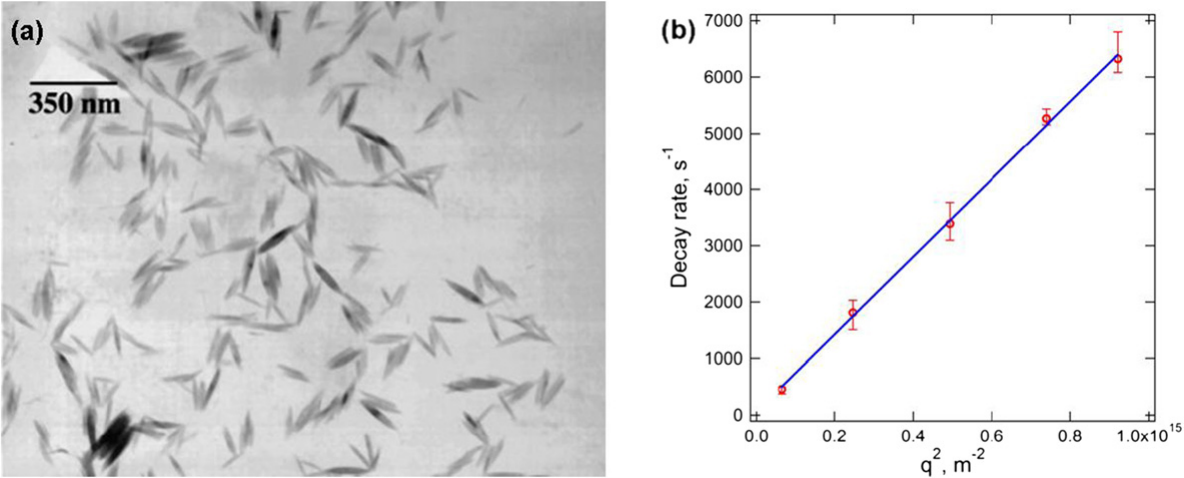
**TEM images and graph of decay rate. (a)** TEM images of β-FeOOH nanorods and **(b)** angle-dependent decay rate *Γ* of the nanorod showing a linear trend. Copyright 2009 Elsevier. Reprinted with permission from [86].

Even though, under most circumstances, a more specialized analytical technique known as depolarized dynamic light scattering is needed to investigate the structural contribution of anisotropic materials [79], it is still possible to extract useful information for rod-like MNPs by conventional DLS measurement [80,81]. For rod-like particles, the decay rate in Equation 6 can be defined as

(14) $$\Gamma =q^{2}D_{T}+6D_{R}$$

where in a plot of *Γ* vs *q*^*2*^, the value of rotational diffusion *D*_R_ can be obtained directly by an extrapolation of *q* to zero and the value of translational diffusion *D*_T_ from the slope of the curve [79]. For rigid non-interacting rods at infinite dilution with an aspect ratio (*L*/*d*) greater than 5, *D*_R_ and *D*_T_ can be expressed using Broersma’s relations [82,83] or the stick hydrodynamic theory [84]. By performing angle-dependent DLS analysis on rod-like β-FeOOH nanorods as shown in Figure 9a, we found that the decay rate is linearly proportional to *q*^*2*^ and passes through the origin (Figure 9b), suggesting that the nanorod motion is dominated by translational diffusion [85]. From Figure 9b, the slope of the graph yields the translational diffusion coefficient, *D*_T_ = 7 × 10^−12^ m^2^/s. This value of *D*_T_ corresponds to an equivalent spherical hydrodynamic diameter of 62.33 nm, suggesting that the DLS results with a single fixed angle of 173° overestimated the true diameter [86]. By taking the length and width of the nanorods as 119.7 and 17.5 nm (approximated from TEM images in Figure 9a), the *D*_T_ calculated by the stick hydrodynamic theory and Broersma’s relationship is 7.09 × 10^−12^ m^2^/s and 6.84 × 10^−12^ m^2^/s, respectively, consistent with the DLS results.

**Figure 10:**
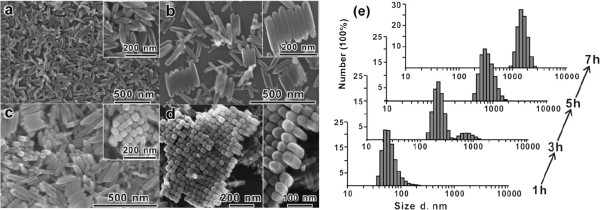
**SEM images of the morphological evolution in the time-dependent experiments. (a)** 1 h, **(b)** 3 h, **(c)** 5 h, and **(d)** 7 h. **(e)** Size distribution of the products obtained in the time-dependent experiments was monitored by DLS with the number averaged. Copyright 2010 American Chemical Society. Reprinted with permission from [87].

Since the β-FeOOH nanorods are self-assembled in a side-by-side fashion to form highly oriented 2-D nanorod arrays and the 2-D nanorod arrays are further stacked in a face-to-face fashion to form the final 3-D layered architectures, DLS can serve as an effective tool to monitor these transient behaviors [87]. Figure 10a depicts the structural changes of self-assembled nanorods over a time course of 7 h. To monitor the in situ real-time behavior of this self-assembly process, DLS was employed to provide the size distribution of the intermediate products that formed in the solution (Figure 10b). The temporal evolution of the detected size from 60 to 70 nm, to dual peaks, to eventually only a single distribution with a peak value of 700 nm indicating that all the building blocks are self-assembled into the large aggregates within the experiment time frame agrees well with the SEM observation (Figure 10a). This kinetic data time scale is involved in the full assembly of anisotropic nanomaterials from single building blocks to 2-D arrays and, eventually, 3-D micron-sized assemblies.

## Conclusion

Dynamic light scattering is employed to monitor the hydrodynamic size and colloidal stability of the magnetic nanoparticles with either spherical or anisotropic structures. This analytical method cannot be employed solely to give feedbacks on the structural information; however, by combining with other electron microscopy techniques, DLS provides statistical representative data about the hydrodynamic size of nanomaterials. In situ, real-time monitoring of MNP suspension by DLS provides useful information regarding the kinetics of the aggregation process and, at the same time, gives quantitative measurement on the size of the particle clusters formed. In addition, DLS can be a powerful technique to probe the layer thickness of the macromolecules adsorbed onto the MNP. However, the interpretation of DLS data involves the interplay of a few parameters, such as the size, concentration, shape, polydispersity, and surface properties of the MNPs involved; hence, careful analysis is needed to extract the right information.

## Competing interests

The authors declare that they have no competing interests.

## Authors’ contributions

JKL synthesized the MNPs, carried out TEM analysis, and drafted the manuscript. SPY carried out DLS measurement and data analysis. HXC carried out DLS measurement and data analysis. SCL participated in the design of the study and drafted the manuscript. All authors read and approved the final manuscript.
